# African immigrants’ favorable preterm birth rates challenge genetic etiology of the Black-White disparity in preterm birth

**DOI:** 10.3389/fpubh.2023.1321331

**Published:** 2024-01-04

**Authors:** Paula Braveman, Katherine Heck, Tyan Parker Dominguez, Kristen Marchi, Wylie Burke, Nicole Holm

**Affiliations:** ^1^Department of Family and Community Medicine, University of California, San Francisco, San Francisco, CA, United States; ^2^Suzanne Dworak-Peck School of Social Work, University of Southern California, Los Angeles, CA, United States; ^3^Department of Bioethics and Humanities, University of Washington, Seattle, Washington, DC, United States

**Keywords:** preterm birth, Black-White disparity in preterm birth, African immigrant, immigrant health, health disparities

## Abstract

**Background:**

We examined over a million California birth records for 2010 through 2021 to investigate whether disparities in preterm birth (PTB) by nativity and race support the widely held but hitherto unsubstantiated belief that genetic differences explain the persistent Black-White disparity in PTB.

**Methods:**

We examined PTB rates and risk ratios among African-, Caribbean-, and U.S.-born Black women compared to U.S.-born White women. Multivariate analyses adjusted for maternal age, education, number of live births, delivery payer, trimester of prenatal care initiation, pre-pregnancy BMI, smoking, and prevalence of poverty in a woman’s residence census tract; and for paternal education.

**Results:**

In adjusted analyses, African-born Black women’s PTB rates were no different from those of U.S.-born White women.

**Discussion:**

The results add to prior evidence making a genetic etiology for the racial disparity in PTB unlikely. If genetic differences tied to “race” explained the Black-White disparity in PTB among U.S.-born women, the African immigrants in this study would have had higher rates of PTB, not the lower rates observed. Multiple explanations for the observed patterns and their implications are discussed. Failure to distinguish causes of PTB from causes of the racial disparity in PTB have likely contributed to erroneous attribution of the racial disparity to genetic differences. Based on the literature, unmeasured experiences of racism, including racism-related stress and adverse environmental exposures, are plausible explanations for the PTB disparity between Black and White U.S.-born women. The favorable birth outcomes of African-born Black immigrants may reflect less exposure to racism during sensitive life periods, e.g., childhood, when they were in African countries, where Black people are in the racial majority.

## Background

Preterm birth (PTB)—delivery prior to 37 weeks of pregnancy—is a health indicator of great importance across the entire life course. It is a strong predictor of infant mortality, childhood disability, and chronic disease in adulthood (1–5). A large disparity in rates of PTB between Black and White women in the United States has been observed for decades (6–8). Some researchers have hypothesized that the Black-White disparity in PTB may reflect genetic differences between the two racial groups (9–11). This hypothesis appears to be based in part on observations in multiple studies that the racial disparity in birth outcomes persists after control for income or education (12, 13).

Convincing evidence of a genetic contribution to the racial disparity in PTB has not been presented, however (14). The persistence of the racial disparity after control for income or education is not evidence of a genetic basis for the disparity. While income and education are important factors for health, they do not capture all potentially important aspects of socioeconomic status, nor effects of racism that do not necessarily operate through socioeconomic pathways. For example, income and education do not capture accumulated wealth, which, because of structural racism (15), varies even more dramatically between Black and White individuals than income or education (16, 17) and could have strong independent effects on PTB (18). Nor do income and education necessarily capture childhood socioeconomic circumstances, which also could have important independent effects on birth outcomes (19–21).

Even if socioeconomic status was measured more comprehensively, the racial disparity in PTB may also reflect unmeasured effects of racism, such as chronic exposure to racism-related stressors (22), and environmental hazards (23) which affect Black persons across the socioeconomic spectrum; in fact, the Black-White disparity in PTB (13) and LBW (24) has been observed to be widest among women with relatively high levels of income and education (25, 26). Chronic stress due to diverse exposures, including racism-related stress, could, over time, trigger inflammatory and immune processes that are known to be involved in PTB (14, 27–29), as well as cardiovascular changes that could influence birthweight-related outcomes (30).

The assumption that genetic causes explain the Black-White disparity in PTB may also reflect a failure to distinguish between causes of PTB and causes of the Black-White disparity in PTB. To explain the disparity, a hypothesized cause would need to have a different prevalence or effect size among Black women compared with White women; to our knowledge, no study of PTB has met those criteria. PTB risk (overall) appears to be influenced by both maternal and fetal genomes (31), and several maternal genetic variants associated with PTB risk have been identified (32). While these account for only 2% of PTB variance, more are likely to be found, given that family and twin studies suggest that genetics may account for 15 to 40% of the variance in PTB; thus, additional genetic contributors to PTB risk are likely to emerge from on-going research. As with other complex traits, the genetic contribution to risk derives from multiple genetic variants, each with small effect, indicating that gene–environment interactions are likely. The wide range of PTB variance estimated to be accounted for by genetic factors likely reflects difficulties in measuring a genetic effect among family members sharing exposure to multiple social and environmental factors. We conclude from the literature not that genetics is unimportant in PTB but that evidence published to date does not support a role for it in explaining racial differences in PTB.

In 1997, Richard David and James Collins published a paper in the New England Journal of Medicine based on examination of Illinois’ 1980–1995 vital records, showing that the birthweight distribution for infants born to Black immigrants from African countries was more similar to that of infants born to U.S.-born White women than that of U.S.-born Black women (33). This pattern also held for low birthweight and very low birthweight. David and Collins (33) did not examine PTB, but noted in a later paper (34) that very low birthweight babies are likely to be preterm. In another investigation, these authors reported that the birthweight distribution of non-Latino Caribbean-born Black mothers was comparable to that of U.S.-born White, but not U.S.-born Black, mothers (24). Caribbean-born Black women also had lower relative risk of moderately low birth weight than U.S.-born Black, but not U.S.-born White women. They hypothesized that these patterns were due to “lifelong minority status” of African American women compared to their Black immigrant counterparts.

Since David and Collins’ landmark 1997 paper, several studies have confirmed their finding of more favorable birthweights among infants of Black African immigrants compared with those of U.S.-born Black women (35, 36), and some studies have documented lower PTB rates among Black Caribbean immigrants compared with U.S.-born Black women (37–39). To our knowledge, however, no previous study has directly compared PTB among Black African immigrants, Black Caribbean immigrants, and White U.S.-born individuals, and explored the implications of that comparison for understanding the role of genetics in the racial disparity in PTB. To that end, we used California birth records to compare rates of PTB among Black immigrants from Africa, Black immigrants from the Caribbean, U.S.-born White women, and U.S.-born Black women who gave birth in California during 2010–2021.

## Methods

Data for these analyses were drawn from California residents’ birth records for 2010 through 2021.^1^ 2021 was the most recent year available, and going back to 2010 yielded sufficient sample size. For the purposes of this study, a sample was constructed consisting of women who reported only Black or White race (i.e., who did not also report another race) and who delivered live singleton infants. Throughout this paper, we use the terms “women” or “mothers” to refer to persons giving birth. We acknowledge, however, that not everyone who experiences pregnancy and gives birth identifies as a woman or mother; our data provide no information on gender identity.

This sample was categorized by self-reported race, ethnicity, and maternal country of birth into the following four groups:

1. African-born Black: single-race, non-Latino Black women born in any African country.
2. U.S.-born Black: single-race, non-Latino Black women born in one of the 50 United States or Washington, D.C. Women born in the U.S. Virgin Islands or Puerto Rico were excluded, based on reasoning that their life experiences likely resembled those of other women born in the Caribbean to an unknown extent. Latino Black women were excluded because their experiences, including experiences of racism, may differ from those of non-Latino Black women.
3. Caribbean-born Black: single-race Black women born in the Caribbean, excluding Puerto Rico or the U.S. Virgin Islands. Because a sizable proportion (15.5%) of Caribbean-born Black women were Latino, Black Latino women were included in this group. Women born in the U.S. Virgin Islands or Puerto Rico were excluded because they may have some experiences similar to those of women born in the 50 United States.
4. U.S.-born White: single-race, non-Latino White women born in one of the 50 United States or Washington, D.C. For comparability with U.S.-born Black women, those born in the U.S. Virgin Islands or Puerto Rico were excluded. Latino White women were excluded because they may have different experiences of racism than White non-Latino women.

Women having multiple births, those identifying as multiracial or members of other racial or ethnic groups, those who were born in countries other than those noted above, and those with missing data on gestational age were excluded. In addition, for consistency across analyses, women with missing data on any covariates in the models were excluded; these covariates included maternal age (<20, 20–24, 25–29, 30–34, 35+), maternal education (less than high school graduate, high school graduate/GED, some college, college graduate or more), trimester of prenatal care initiation (first, second, third or none), number of live births (1, 2–4, 5 or more), delivery payer [private insurance, Medi-Cal (California’s Medicaid program), other, uninsured], height and weight [calculated as body mass index <18.5 (underweight), 18.5–24.9 (healthy weight), 25–29.9 (overweight), 30 or more (obese)], smoking during pregnancy (yes or no), and poverty rate in the census tract of residence (<10%, 10–19%, 20–29, 30%+). Paternal education also was included and categorized the same way as maternal education, but due to a higher percentage of missing data than for other variables, “missing” was included as an additional category for paternal education. Overall, 9.6% of women with live births who fit within one of the four groups of primary analytical interest were excluded due to missing data for at least one covariate; 1,402,606 records were included in final analyses (Table 1).

**Table 1:** Sample.

Group	Sample size, 2010–2021
African-born Black	21,705
U.S.-born Black	210,759
Caribbean-born Black	3,202
U.S.-born White	1,166,940

Data were analyzed using SAS® 9.4. (40). Percentages and 95% confidence intervals were calculated for sample characteristics in each group. Risk ratios and 95% confidence intervals were calculated based on Poisson regression models (41) for preterm birth. Multivariate analyses assessed whether differences in PTB among the groups of interest persisted after adjusting for the above factors. U.S.-born White women were the reference group because the study’s focus was on the Black-White disparity in PTB.

## Results

Maternal and paternal characteristics varied across the four groups (Table 2). Countries of origin for African- and Caribbean-born women are listed in Supplementary Tables S1, S2. U.S.-born Black women were younger than women in the other groups. Education levels were highest among U.S.-born White and African-born Black women. About 43% of African-born Black women were insured by Medi-Cal, as were 56% of U.S.-born Black women, 39% of Caribbean-born Black women, and 21% of U.S.-born White women. Levels of underweight were similar across the four groups (3.4–3.7%), while U.S.-born Black women were more likely to be obese than other women. Black and White U.S.-born women were more likely to smoke during pregnancy (3.6 and 3.7%, respectively) than were African-born or Caribbean-born women (0.2%). U.S.-born Black women were more likely to live in high-poverty census tracts (25.3%) than were members of the other groups (5.3–12.2%).

**Table 2:** Characteristics of the sample.

Characteristic	African-born Black		U.S.-born Black		Caribbean-born Black		U.S.-born White	
Total	21,705		210,759		3,202		1,166,940	
	%	Confidence interval	%	Confidence interval	%	Confidence interval	%	Confidence interval
**Maternal age (%)**								
10–19	0.4	(0.3–0.5)	8.3	(8.2–8.5)	1.4	(1.0–1.8)	2.4	(2.4–2.4)
20–24	5.6	(5.3–5.9)	26.8	(26.7–27.0)	10.7	(9.7–11.8)	12.7	(12.7–12.8)
25–29	23.3	(22.8–23.9)	28.3	(28.1–28.5)	24.8	(23.3–26.4)	26.3	(26.2–26.4)
30–34	36.6	(36.0–37.2)	22.0	(21.8–22.2)	31.9	(30.3–33.6)	34.9	(34.8–35.0)
35 or older	34.1	(33.4–34.7)	14.6	(14.4–14.7)	31.2	(29.6–32.8)	23.7	(23.6–23.8)
**Maternal education (%)**								
Less than high school graduate	6.2	(5.9–6.5)	12.3	(12.1–12.4)	7.3	(6.4–8.2)	3.7	(3.6–3.7)
High school graduate/GED	20.1	(19.6–20.6)	34.7	(34.5–34.9)	22.3	(20.8–23.8)	18.5	(18.5–18.6)
Some college	29.5	(28.9–30.1)	37.4	(37.2–37.6)	34.9	(33.2–36.5)	29.2	(29.1–29.3)
College graduate	44.3	(43.6–45.0)	15.7	(15.5–15.8)	35.6	(33.9–37.3)	48.6	(48.5–48.7)
**Paternal education (%)**								
Less than high school graduate	3.3	(3.1–3.5)	8.5	(8.4–8.6)	5.7	(4.9–6.6)	3.8	(3.8–3.8)
High school graduate/GED	15.4	(15.0–15.9)	34.5	(34.3–34.7)	21.7	(20.3–23.2)	23.3	(23.2–23.4)
Some college	23.6	(23.1–24.2)	25.4	(25.3–25.6)	28.8	(27.2–30.4)	27.0	(27.0–27.1)
College graduate	50.0	(49.3–50.7)	10.1	(10.0–10.2)	31.3	(29.7–32.9)	41.3	(41.2–41.4)
Missing	7.7	(7.3–8.0)	21.4	(21.2–21.6)	12.5	(11.4–13.7)	4.5	(4.5–4.6)
**Delivery payer (%)**								
Medi-Cal	43.3	(42.6–43.9)	55.5	(55.3–55.7)	39.4	(37.7–41.1)	21.4	(21.3–21.5)
Private	45.0	(44.4–45.7)	35.3	(35.1–35.5)	43.1	(41.4–44.9)	70.9	(70.8–71.0)
Other	5.3	(5.0–5.7)	8.0	(7.9–8.1)	15.0	(13.8–16.3)	5.5	(5.4–5.5)
Uninsured	6.3	(6.0–6.7)	1.2	(1.1–1.2)	2.5	(2.0–3.1)	2.2	(2.2–2.2)
**Number of live births (%)**								
First birth	35.6	(35.0–36.3)	39.7	(39.5–40.0)	41.1	(39.4–42.9)	44.5	(44.4–44.6)
2nd-4th birth	58.9	(58.3–59.6)	53.1	(52.9–53.3)	55.7	(54.0–57.5)	52.8	(52.7–52.9)
5 births or more	5.5	(5.2–5.8)	7.2	(7.1–7.3)	3.2	(2.6–3.8)	2.7	(2.7–2.7)
**Trimester of prenatal care initiation (%)**								
First	79.5	(78.9–80.0)	80.5	(80.3–80.6)	83.9	(82.6–85.2)	88.9	(88.8–88.9)
Second	15.2	(14.7–15.7)	15.1	(14.9–15.2)	11.3	(10.2–12.4)	9.1	(9.0–9.1)
Third or none	5.3	(5.0–5.6)	4.5	(4.4–4.5)	4.8	(4.1–5.6)	2.0	(2.0–2.1)
**Pre-pregnancy body mass index (%)**								
Underweight (BMI <18.5)	3.7	(3.4–3.9)	3.6	(3.5–3.7)	3.4	(2.8–4.1)	3.6	(3.5–3.6)
Healthy weight (BMI 18.5–24.9)	45.4	(44.7–46.0)	36.1	(35.9–36.3)	44.9	(43.2–46.6)	53.6	(53.5–53.7)
Overweight (BMI 25.0–29.9)	32.1	(31.5–32.8)	26.4	(26.2–26.6)	30.3	(28.7–31.9)	23.5	(23.5–23.6)
Obese (BMI > =30.0)	18.8	(18.3–19.4)	33.9	(33.7–34.1)	21.5	(20.1–23.0)	19.3	(19.3–19.4)
**Smoking during pregnancy (%)**	0.2	(0.1–0.3)	3.6	(3.5–3.7)	0.2	(0.1–0.5)	3.7	(3.7–3.8)
**Census tract poverty (%)**								
Low poverty (<10%)	34.1	(33.5–34.7)	20.2	(20.0–20.4)	35.4	(33.7–37.0)	51.4	(51.3–51.5)
10–19%	35.3	(34.7–36.0)	30.2	(30.0–30.4)	34.5	(32.9–36.2)	32.2	(32.1–32.3)
20–29%	18.4	(17.9–18.9)	24.3	(24.1–24.5)	19.6	(18.2–21.0)	11.1	(11.1–11.2)
High poverty (> = 30%)	12.2	(11.8–12.6)	25.3	(25.2–25.5)	10.6	(9.5–11.7)	5.3	(5.3–5.4)

Among the four groups, U.S.-born Black women had the highest rate of preterm birth (10.1%), while U.S.-born White women had the lowest rate (5.7%) (Table 3). The rate for African-born Black women was 6.2%.

**Table 3:** Preterm birth rates.

Birth outcomes	African-born Black	U.S.-born Black	Caribbean-born Black	U.S.-born White
Preterm birth (%)	6.2 (5.8–6.5)	10.1 (10.0–10.2)	8.4 (7.5–9.4)	5.7 (5.7–5.8)

The main results from multivariate models are shown in Table 4; results for all covariates are shown in Supplementary Tables S1, S2. In unadjusted models, African-born Black women had slightly but statistically significantly higher risk of preterm birth than U.S.-born White women [risk ratio 1.08, 95% confidence interval (CI) 1.02–1.14]. After adjustment for covariates, there was no longer a significant difference in the incidence of preterm birth between African-born Black and U.S.-born White women (risk ratio, 1.02; 95% CI 0.97–1.08). After adjustment for covariates, however, Caribbean-born Black women continued to have a higher risk for PTB than U.S.-born White women. U.S.-born Black women had the highest risk of PTB compared to U.S.-born White women (risk ratio 1.52, 95% CI 1.49–1.54).

**Table 4:** Multivariate models for preterm birth (results for full model with all covariates are in Supplemental Tables S1, S2).

	African-born Black	U.S.-born Black	Caribbean-born Black	U.S.-born White
Unadjusted				
Risk ratio and 95% confidence interval	1.08 (1.02–1.14)	1.77 (1.74–1.79)	1.46 (1.30–1.65)	(ref.)
Adjusted*				
Risk ratio and 95% confidence interval	1.02 (0.97–1.08)	1.52 (1.49–1.54)	1.33 (1.18–1.50)	(ref.)

*Adjusting for maternal age, maternal education, paternal education, number of live births, delivery payer, trimester of prenatal care initiation, body mass index, smoking during pregnancy, census tract poverty.

To examine whether results would change if Black Latino women were included, sensitivity analyses were performed with and without Black Latino women in all three nativity groups of Black women. Results were very similar for African-born and U.S.-born Black women whether or not Latino women were included in adjusted models. Risk ratios for Caribbean-born Black women were somewhat higher when Latino were excluded [adjusted risk ratio 1.42 (1.25–1.61)] (not displayed).

Because paternal education had so much missing data, sensitivity analyses were computed including and excluding paternal education as a covariate in models. Results did not change whether models included or excluded paternal education, or whether individuals missing paternal education were included in a missing-paternal-education category or excluded from models (not displayed). Results also did not change when additional sensitivity analyses were conducted treating age as a continuous rather than categorical variable, or when splitting the college-educated group into college graduates and those with post-graduate education (not displayed).

## Discussion

This study confirmed the findings of previous research showing that PTB rates among Black African immigrants are far more favorable than rates among U.S.-born Black women. The main objective of this study, however, was not to confirm the lower PTB rates of Black immigrants relative to U.S.-born Black individuals, which has been well documented, but to compare the PTB rates of Black African and Caribbean immigrants with those of White U.S.-born individuals, and to interpret the implications for understanding the potential role of genetics in the large and persistent disparity in PTB between U.S.-born Black and White women.

In this large population-based sample (n = 1,402,606) of live births in California, where one in every nine U.S. births occurs (42), U.S.-born and Caribbean-born Black women had higher PTB rates than US-born White women and African-born Black women, even after adjusting for differences in characteristics such as age, parity, maternal education, paternal education, delivery payer, trimester of prenatal care initiation, and pre-pregnancy BMI; however, there was no PTB disparity between Black African immigrants and U.S.-born White women.

A number of reasons have been offered for the favorable PTB outcomes of Black African immigrants. The well-documented “healthy immigrant” effect posits that foreign-born individuals are generally in better health prior to immigrating (43, 44), and bring with them healthier behaviors and stronger social support that may buffer the stress of transitioning to a new environment (37, 45). While controlling for maternal education has not accounted for the nativity disparity in PTB, two studies have found paternal education to play a significant role (46, 47). Ekeke et al. (46) attributed 15% of the maternal nativity disparity to low paternal educational attainment among the U.S.-born, hypothesizing that increased paternal educational attainment may reflect increased social and financial support of the mother. Unique to African-born women specifically may be the role of experiences of discrimination. Dominguez et al. (48) found the prevalence of self-reported discrimination, while notable among all Black groups, to be lowest among African-born women, and comparable between US-born and Caribbean-born women. African-born Black women emigrated from countries in which they were in the racial majority and thus likely experienced less or less severe racial discrimination. A life-course perspective emphasizes the impact on birth outcomes of exposures not only during pregnancy, but also throughout life leading up to pregnancy (21). Collins et al. (49) found that the infants born to US-born daughters of immigrant Black women had lower average birthweights than the previous generation, indicating a loss of the “reproductive advantage” of the prior (immigrant) generation. This and other research (22) has suggested that there are features of the U.S. social context, including a range of experiences of racism and its consequences, that are toxic to Black women’s childbearing health (20, 50, 51).

Similarly, we can only speculate about why Caribbean-born Black women had lower rates of PTB than their U.S.-born Black counterparts, but higher rates than those of African-born Black women or U.S.-born White women. The characteristics (e.g., maternal and paternal education, delivery payer, census tract poverty rate, weight) of Caribbean-born women were more favorable than those of U.S.-born Black women, but not as favorable as those of African-born Black women or U.S.-born White women. While our analyses controlled for a number of important markers of risk, Caribbean-born Black women may be at higher risk for adverse birth outcomes than African-born immigrants and US-born White women due to unmeasured differences in risk characteristics (14). Caribbean-born Black women may be at higher risk than African-born Black women because Caribbean countries share with the United States a long history of European colonization and slavery (48, 52). That history may have left an enduring legacy of racism, including structural racism, with consequences including pervasive racism-related stress and disadvantage. Dominguez et al. (48) found that Caribbean women’s perceptions of racism were more similar to those of U.S.-born than African-born Black women. Many studies have linked racism-related stress to the Black-White disparity in PTB and have concluded that environmental injustice and other manifestations of structural racism likely contribute; plausible physiologic pathways and mechanisms have been identified (14). On the other hand, better outcomes of Caribbean-born Black women compared with U.S.-born Black women may reflect the fact that throughout most of the Caribbean region, Black people constitute the large majority of the population. In 10 of the 13 sovereign states of the Caribbean, over 70% of the population is of African descent; in 9 of those 13 nations, over 80% of the population is of African descent (53). Historically, many political leaders in the region have been and continue to be of African descent (54). The nature, extent, and/or depth of racism experienced by Caribbean-born Black women and the U.S.-born Black women descendants of American chattel slavery may differ in important ways (48).

While the findings of this and other studies make a genetic etiology of the Black-White disparity in PTB unlikely, they do not rule out epigenetic phenomena or complex interactions between social and genetic contributors to PTB. Genetic research can help to define biological pathways and physiological mechanisms underlying gestational length (31). As genetic contributors to PTB risk are identified, they may also enable studies of gene–environment interactions that could inform interventions to reduce PTB disparities. It is important to underscore, however, that genetic contributors to PTB are not the same as genes tied to “race” as a cause of racial disparities in PTB. Unproven or disproven assumptions about race-based genetic differences as a cause of racial disparities in health outcomes have often been used, sometimes unwittingly, in ways that justify and reinforce racism and White supremacy. These assumptions confuse superficial secondary physical characteristics, such as skin color and hair texture, with fundamental biological differences such as intelligence, perception of pain, or susceptibility to chronic disease; research, however, has not found these to be correlated (55–58).

It is important to be aware that, given the legacy of racism in the U.S., the issue of a genetic etiology for the Black-White disparity in PTB is a particularly sensitive one. The concept of physically distinct superior and inferior “races” emerged in the seventeenth century with the trans-Atlantic slave trade; it was used to justify the enslavement of human beings (59). This history and clear evidence to this day of ongoing White supremacy and oppression of minoritized populations are the essential context for appreciating the importance of understanding that race is a biologically discredited, although highly significant social construct (60, 61).

Strengths of this study include the large sample size and the use of sensitivity analyses that tested whether differences in sample exclusions or inclusions or whether different ways of specifying variables would make a difference in the conclusions. A limitation of this study is that the data do not include genomic markers and thus cannot establish the degree of genetic similarity between the U.S.-born Black women and African immigrants in the sample. Nevertheless, the similarity in PTB rates among African immigrants and U.S.-born White women, along with the striking difference in rates of PTB between African immigrants and U.S.-born Black women argue against a “race”-based genetic cause for the racial disparity in PTB seen in the United States. David and Collins (33) estimated that U.S.-born Black women on average had a 20–30 percent admixture of European genetic material, based on geographic ancestry markers. They reasoned that if the disparity in birthweight among U.S.-born Black and White individuals were genetically based, the risk for low birthweight among U.S.-born Black women compared with that of African immigrants would have been lower, not higher, as was observed, given that the African immigrants would have far less, if any, European genetic admixture than their U.S.-born counterparts. The same reasoning applies to our study of PTB: if genetic differences tied to “race” explained the large and persistent Black-White disparity in PTB among U.S.-born women, the African immigrants in our study would have had higher rates of PTB, not the lower rates observed.

As with all research, the possibility of residual confounding by unmeasured differences cannot be ruled out; it is reassuring, however, that our results were quite robust to many sensitivity analyses. The major limitation of this study is the lack of information on the length of time that immigrants had lived in the U.S. In addition, we lacked information on childhood experiences, including socioeconomic and other social conditions that could have major impact on later reproductive outcomes. Another limitation is the absence of genomic information. Furthermore, in demonstrating that it is unlikely that the Black-White disparity in PTB among U.S.-born individuals reflects genetic differences, this study does not identify the cause(s) of that disparity, which are not definitively known. Most scholars agree that the causation is likely to be complex and multifactorial.

Many downstream and midstream factors are biologically plausible as contributors to the racial disparity in PTB. For example, chronic stress could affect PTB through neuroendocrine and immune mechanisms leading to inflammation and immune dysfunction (28); stress could alter an individual’s microbiota, immune response to infection, chronic disease risks, and behaviors, and trigger epigenetic changes influencing PTB risk (29, 62, 63). As an upstream factor, racism in multiple forms has repeatedly been linked with plausible midstream or downstream factors, including socioeconomic disadvantage, stress, and toxic exposures (14). To our knowledge, racism is the only factor that directly or indirectly could explain the observed racial disparities in multiple plausible midstream/downstream causes and the observed social patterning. Historical and contemporary structural racism could explain the racial disparities in socioeconomic opportunities that differentially expose so many African Americans to lifelong financial stress and associated health-harming conditions (15). Segregation places Black women in stressful surroundings and exposes them to environmental hazards (64). Race-based discriminatory treatment is a pervasive stressor for Black women of all socioeconomic levels (65). The results suggest that the nature and/or severity of racism may vary for Black women in different nativity groups, along with resilience to its health-harming effects; timing of exposure to racism may matter, for example, during childhood (as among the U.S.-born) versus adulthood (as among many immigrants) (48). Neuroscience has revealed that chronic stress during childhood has particularly toxic and often lifelong adverse health effects (66). Many scholars have concluded that racism is a highly plausible, major upstream contributor to the Black-White disparity in PTB through multiple pathways and biological mechanisms (14, 19, 67–69). Research to elucidate the social causes of the Black-White disparity in PTB should be a high priority. Action against racism need not await definitive answers, however. While much is unknown, existing knowledge and the core values of equity and justice support addressing racism now in efforts to eliminate the racial disparity in PTB and many other important health outcomes.

## Data availability statement

Publicly available datasets were analyzed in this study. This data can be found at: https://www.cdph.ca.gov/Programs/CHSI/Pages/Data-Applications.aspx.

## Author contributions

PB: Writing – original draft, Writing – review & editing. KH: Writing – original draft, Writing – review & editing. TD: Writing – original draft, Writing – review & editing. KM: Writing – original draft, Writing – review & editing. WB: Writing – original draft, Writing – review & editing. NH: Writing – original draft, Writing – review & editing.

## Footnotes

^1^California Birth Statistical Master File, 2010–2017, and California Comprehensive Master Birth File, 2018–2021.
