# Supplementary material for: African immigrants’ favorable preterm birth rates challenge genetic etiology of the Black-White disparity in preterm birth
Source: Front Public Health. 2024 Jan 4;11:1321331. doi: 10.3389/fpubh.2023.1321331 (PMC10794556; doi:10.3389/fpubh.2023.1321331)
Supplement: Supplementary file 1 [file Data_Sheet_1.docx]

**African Immigrants’ Favorable Preterm Birth Rates Challenge Genetic Etiology of the Black-White Disparity in Preterm Birth**

Paula Braveman^1^, Katherine Heck^1^, Tyan Parker Dominguez^2^, Kristen Marchi^1^, Wylie Burke^3^, Nicole Holm^1^

^1^Department of Family and Community Medicine, University of California, San Francisco, San Francisco, CA, USA

^2^Suzanne Dworak-Peck School of Social Work, University of Southern California, Los Angeles, CA, USA

^3^Department of Bioethics and Humanities, University of Washington, Seattle, Washington, USA

*** Correspondence:**Paula Braveman
paula.braveman@ucsf.edu

Keywords: Preterm birth; Black African immigrants; Genetics; Racism; Health disparities; Black-white disparity in preterm birth

## Supplemental Tables

**Supplemental Table 1. Countries of birth for African-born and Caribbean-born women**

| **Table 1. Countries of birth for African-born and Caribbean-born women** | |
| --- | --- |
|  |  |
| **African-born** | **Caribbean-born** |
| Algeria | Anguilla |
| Angola | Antigua or Barbuda |
| Benin | Bahamas |
| Botswana | Barbados |
| Burkina Faso | British Virgin Islands |
| Burundi | Cayman Islands |
| Cameroon | Cuba |
| Cape Verde | Dominica |
| Central African Republic | Dominican Republic |
| Chad | Grenada |
| Comoros | Guadeloupe |
| Cote d'Ivoire | Haiti |
| Democratic Republic of the Congo | Jamaica |
| Djibouti | Martinique |
| Egypt | Montserrat |
| Equatorial Guinea | Saint Kitts or Nevis |
| Eritrea | Saint Lucia |
| Ethiopia | Saint Vincent or Grenadines |
| Gabon | Trinidad or Tobago |
| Gambia | Turks Islands or Caicos Islands |
| Ghana |  |
| Guinea |  |
| Guinea-Bissau |  |
| Kenya |  |
| Lesotho |  |
| Liberia |  |
| Libya |  |
| Madagascar |  |
| Malawi |  |
| Mali |  |
| Mauitania |  |
| Mauritius |  |
| Morocco |  |
| Mozambique |  |
| Namibia |  |
| Niger |  |
| Nigeria |  |
| Republic of the Congo |  |
| Rwanda |  |
| Sao Tome and Principe |  |
| Senegal |  |
| Seychelles |  |
| Sierra Leone |  |
| Somalia |  |
| South Africa |  |
| South Sudan |  |
| Sudan |  |
| Swaziland |  |
| Tanzania |  |
| Togo |  |
| Tunisia |  |
| Uganda |  |
| Western Sahara |  |
| Zambia |  |
| Zimbabwe |  |

**Supplemental Table 2. Adjusted model (full results for all covariates)**

| **Variable** | **Category** | **Risk ratio** | **95% Confidence Interval** |
| --- | --- | --- | --- |
| Race and nativity | Black, African born | 1.02 | (0.97-1.08) |
|  | Black, Caribbean born | 1.33 | (1.18-1.50) |
|  | Black, US born | 1.52 | (1.49-1.54) |
|  | White, US born | ref. |  |
| Maternal age | <20 | 0.86 | (0.83-0.89) |
|  | 20-24 | 0.91 | (0.89-0.93) |
|  | 25-29 | ref. |  |
|  | 30-34 | 1.14 | (1.12-1.16) |
|  | 35+ | 1.43 | (1.40-1.46) |
| Maternal education | Less than high school graduate | 1.33 | (1.29-1.38) |
|  | High school graduate/GED | 1.20 | (1.17-1.23) |
|  | Some college | 1.18 | (1.16-1.21) |
|  | College graduate | ref. |  |
| Paternal education | Less than high school graduate | 1.29 | (1.25-1.34) |
|  | High school graduate/GED | 1.18 | (1.16-1.21) |
|  | Some college | 1.13 | (1.10-1.15) |
|  | College graduate | ref. |  |
|  | Missing | 1.35 | (1.31-1.39) |
| Trimester of prenatal care initiation | 1st trimester | ref. |  |
|  | 2nd trimester | 0.91 | (0.89-0.93) |
|  | 3rd trimester or none | 1.35 | (1.31-1.40) |
| Number of live births | First birth | 1.25 | (1.23-1.26) |
|  | 2nd-4th birth | ref. |  |
|  | 5th birth or more | 1.33 | (1.29-1.37) |
| Delivery payer | Private | ref. |  |
|  | Medi-Cal | 1.09 | (1.07-1.11) |
|  | Other | 0.98 | (0.95-1.01) |
|  | None / Self pay | 0.66 | (0.62-0.70) |
| Pre-pregnancy body mass index | Underweight | 1.33 | (1.29-1.37) |
|  | Healthy weight | ref. |  |
|  | Overweight | 1.01 | (0.99-1.03) |
|  | Obese | 1.17 | (1.15-1.19) |
| Smoking during pregnancy | Yes | 1.36 | (1.32-1.40) |
|  | No | ref. |  |
| Percent below poverty in census tract | <10% | ref. |  |
|  | 10-19% | 1.01 | (1.00-1.03) |
|  | 20-29% | 1.03 | (1.01-1.05) |
|  | 30%+ | 1.06 | (1.04-1.09) |
